# Identifying Electronic Health Record Contributions to Diagnostic Error in Ambulatory Settings Through Legal Claims Analysis

**DOI:** 10.1001/jamanetworkopen.2023.8399

**Published:** 2023-04-14

**Authors:** Seth A. Krevat, Sunil Samuel, Christian Boxley, Vishnu Mohan, Dana Siegal, Jeffrey A. Gold, Raj M. Ratwani

**Affiliations:** 1 MedStar Health National Center for Human Factors in Healthcare, Washington, DC; 2 Department of Medical Informatics and Clinical Epidemiology, Oregon Health & Science University, Portland; 3 CRICO, Boston, Massachusetts

## Abstract

This qualitative study analyzes closed legal claims data to determine whether problems with electronic health records are associated with diagnostic errors, in which part of the diagnostic process errors occur, and the specific types of errors that occur.

## Introduction

Electronic health records (EHRs), which are used by most health care facilities in the US, are the central repository for clinical information required for diagnostic accuracy. Clinicians require timely access to appropriate clinical information, which should be readily available and displayed in ways that support the diagnostic process. However, numerous EHR issues, including software malfunctions, interoperability challenges, and usability issues, have been identified and may be associated with patient harm.^1,2,3^ Through analysis of closed legal claims data, we sought to identify whether problems with EHRs are associated with diagnostic errors, in which part of the diagnostic process errors occur, and the specific types of errors that occur.

## Methods

This qualitative study was approved by the Oregon Health Sciences University institutional review board and was conducted and reported in accordance with the Standards for Reporting Qualitative Research (SRQR) reporting guideline. Because the study used deidentified claims, this study had a waiver of informed consent in accordance with 45 CFR §46.

Claims were retrieved from the CRICO malpractice insurance database, which represents more than 550 inpatient and outpatient health care facilities. Claims were coded based on a proprietary taxonomy defining the allegation type and contributing factors. Claims in ambulatory settings with a loss year between 1987 and 2020 that were closed between January 2015 and September 2021 with the allegation categories of diagnosis-related and EHR-related contributing factors were qualitatively analyzed. Two physicians (S.A.K., S.S.) reviewed each claim summary to determine the presence of a diagnostic error, the stage of the diagnostic process in which the error occurred, the type of error, and the outcome of the error. If the 2 physicians could not reach consensus on analysis, a third physician (J.A.G.) reviewed the claim. Because this is a descriptive study, no statistical analysis was conducted. The presence of a diagnostic error was defined as inaccurately determining which disease or condition explains a patient's signs or symptoms. The stage of the diagnostic process was categorized as 1 or more of the following: testing stage, assessment stage, or referral stage^4^ (see the eAppendix in Supplement 1 for definitions). Error types within each diagnostic process stage were categorized as 1 or more of the following^4^: order, execution, notification, data interpretation, documentation, and communication (see the eAppendix in Supplement 1 for definitions). Error outcomes were categorized as 1 of the following: missed or delayed diagnosis, wrong diagnosis, or other.

## Results

There were 238 claims coded as diagnosis related with an EHR contributing factor. After physician review, 199 claims were confirmed to be diagnosis related. Of the 199 diagnosis-related claims, the EHR was considered a potential contributor to the diagnostic error in 122 claims (61.3%). The contribution of the EHR was unable to be determined for 33 claims (13.9%), and EHR was determined to be noncontributory for 44 claims (18.5%) . Table 1 shows the claim location information, and Table 2 shows responsible service information.

**Table 1.** Claim Locations

Location	Claims, No. (%)	
	Total diagnosis related with an EHR contributing factor (N = 238)	Confirmed by physician review as diagnosis related with an EHR contributing factor (n = 122)
Outpatient physician office	130 (54.6)	63 (51.6)
Emergency or urgent care	48 (20.2)	21 (17.2)
Radiology	24 (10.1)	21 (17.2)
Other	22 (9.2)	9 (7.4)
Operating room or procedural areas	5 (2.1)	3 (2.5)
Pathology or clinical laboratory	5 (2.1)	4 (3.3)
Intensive care unit (surgical intensive care unit, medical intensive care unit, critical care unit)	3 (1.3)	0
Therapy or rehabilitation	1 (0.4)	1 (0.8)

Abbreviation: EHR, electronic health record.

**Table 2.** Type of Service Responsible for Claim

Type of service	Claims, No. (%)	
	Total diagnosis related with an EHR-contributing factor (N = 238)	Confirmed by physician review as diagnosis related with an EHR- contributing factor (n = 122)
Family medicine	33 (13.9)	18 (14.8)
Emergency medicine	31 (13.0)	12 (9.8)
Radiology	30 (12.6)	25 (20.5)
Internal medicine	29 (12.2)	15 (12.3)
Gynecology	12 (5.0)	8 (6.6)
Gastroenterology	11 (4.6)	6 (4.9)
Medical hospitalist	10 (4.2)	4 (3.3)
Cardiology	8 (3.4)	3 (2.5)
Dermatology	6 (2.5)	0
Orthopedic	5 (2.1)	4 (3.3)
Pathology	5 (2.1)	4 (3.3)
Pediatrics	5 (2.1)	0
Nursing	5 (2.1)	2 (1.6)
Pulmonary disease	4 (1.7)	2 (1.6)
General surgery	4 (1.7)	2 (1.6)
Urology surgery	4 (1.7)	1 (0.8)
Obstetrics	4 (1.7)	2 (1.6)
Neurology	4 (1.7)	1 (0.8)
Clinical laboratory	3 (1.3)	3 (2.5)
Otolaryngology (no plastic)	3 (1.3)	0
Neurosurgery	3 (1.3)	1 (0.8)
Ophthalmology	3 (1.3)	2 (1.6)
Hand surgery	2 (0.8)	0
Psychiatry	2 (0.8)	1 (0.8)
Dentistry	1 (0.4)	1 (0.8)
Integrative medicine	1 (0.4)	0
Midwifery	1 (0.4)	1 (0.8)
Interventional neuroradiology	1 (0.4)	0
Chiropractic	1 (0.4)	0
Plastic surgery	1 (0.4)	0
Interventional cardiology	1 (0.4)	1 (0.8)
Interventional radiology	1 (0.4)	0
Medicine, other	1 (0.4)	0
Cardiac surgery	1 (0.4)	1 (0.8)
Hematology	1 (0.4)	1 (0.8)
Other allied health care	1 (0.4)	1 (0.8)

Abbreviation: EHR, electronic health record.

Analyses of the diagnostic stage for the 122 claims with EHR as a potential contributor found that 112 claims (91.8%) involved the testing stage, 26 claims (21.3%) involved the assessment stage, and 3 claims (2.5%) involved the referral stage. The most common error types included 62 execution errors (50.8%), 56 ordering errors (45.9%), and 56 data interpretation errors (45.9%) There were also 8 documentation errors (6.6%) and 7 communication errors (5.7%). The outcomes of these 122 claims included 87 (71.3%) missed or delayed diagnoses, 33 (27.0%) wrong diagnoses and 2 (1.7%) claims where the outcome was unclear. Of the 122 claims with EHR as a potential contributor, 91 (74.6%) claims had a judgment or settlement noted.

## Discussion

The findings of this qualitative study illustrate the central role of the EHR for supporting the diagnostic process, and the potential for EHR issues such as suboptimal design or clinician use to facilitate diagnostic errors. The reliance on the claim summary for review, as opposed to the entire legal medical record, limits our findings. Furthermore, any issues that did not result in a claim are not part of our analysis and the analysis does not account for EHR adoption and optimizations over time. These results highlight the importance of the continued work needed to optimize EHR design, interoperability, training, and safety surveillance to reduce diagnostic errors. Additionally, to inform improvement efforts, more granular reporting taxonomies are needed to better understand the specific EHR issues that may be contributing to diagnostic errors.

## Notes

Supplement 1. **eAppendix.** Supplemental Methods

Supplement 2.
Data Sharing Statement

## References

[1] Howe JL, Adams KT, Hettinger AZ, Ratwani RM. Electronic health record usability issues and potential contribution to patient harm. JAMA. 2018;319(12):1276-1278. doi:10.1001/jama.2018.1171 29584833 PMC5885839

[2] Apathy NC, Howe JL, Krevat SA, . Electronic health record legal settlements in the US since the 2009 Health Information Technology for Economic and Clinical Health Act. JAMA Health Forum. 2022;3(11):e223872. doi:10.1001/jamahealthforum.2022.3872 36367740 PMC9652746

[3] Pacheco TB, Hettinger AZ, Ratwani RM. Identifying potential patient safety issues from the federal electronic health record surveillance program. JAMA. 2019;322(23):2339-2340. doi:10.1001/jama.2019.17242 31846009 PMC6990822

[4] Schiff GD, Hasan O, Kim S, . Diagnostic error in medicine: analysis of 583 physician-reported errors. Arch Intern Med. 2009;169(20):1881-1887. doi:10.1001/archinternmed.2009.333 19901140

