# Supplementary material for: Identifying Electronic Health Record Contributions to Diagnostic Error in Ambulatory Settings Through Legal Claims Analysis
Source: JAMA Netw Open. 2023 Apr 14;6(4):e238399. doi: 10.1001/jamanetworkopen.2023.8399 (PMC10105306; doi:10.1001/jamanetworkopen.2023.8399)
Supplement: Supplement 1. — eAppendix. Supplemental Methods [file jamanetwopen-e238399-s001.pdf]

Supplemental Online Content

Krevat SA, Samuel S, Boxley C, et al. Identifying electronic health record contributions to diagnostic error in ambulatory settings through legal claims analysis. *JAMA Netw Open*. 2023;6(4):e238399. doi:10.1001/jamanetworkopen.2023.8399

eAppendix. Supplemental Methods

This supplemental material has been provided by the authors to give readers additional information about their work.

© 2023 Krevat SA et al. *JAMA Network Open.*

eAppendix. Supplemental Methods

Definition of stages of diagnostic process:

Testing: Issues related to timely ordering of appropriate tests, performing testing correctly, and clinician processing of test results.

Assessment: Issues involved considering the correct diagnosis, conducting the correct physical assessment, and correct interpretation of tests.

Referral: Issues related to adequate access to care, timely referral for consultation, and consultation follow-up.

Definitions of specific type of error within each diagnostic process stage

Order: Failure or delay in ordering the appropriate test.

Execution/Notification:. Failing to complete, delaying, or making an error during a test. Failing to notify a clinician of the results of a test.

Interpretation: Incorrectly interpreting a test or failing or delayed follow-up of tests.

Documentation: Entering the wrong information.

Communication:. Failing to communicate results to a patient or follow-up after consultation.

© 2023 Krevat SA et al. *JAMA Network Open.*
