# Supplementary material for: Identifying Electronic Health Record Contributions to Diagnostic Error in Ambulatory Settings Through Legal Claims Analysis
Source: JAMA Netw Open. 2023 Apr 14;6(4):e238399. doi: 10.1001/jamanetworkopen.2023.8399 (PMC10105306; doi:10.1001/jamanetworkopen.2023.8399)
Supplement: Supplement 2. — Data Sharing Statement [file jamanetwopen-e238399-s002.pdf]

Data Sharing Statement

Krevat. Identifying Electronic Health Record Contributions to Diagnostic Error in Ambulatory Settings Through Legal Claims Analysis. *JAMA Netw Open*. Published April 14, 2023. doi:10.1001/jamanetworkopen.2023.8399

Data
Data available: No

Additional Information
Explanation for why data not available: Data were derived from confidentrial medical malpractice files and these can not be shared
